# New Evidence on the Impact of Antithrombotics in Patients Submitted to Small Bowel Capsule Endoscopy for the Evaluation of Obscure Gastrointestinal Bleeding

**DOI:** 10.1155/2014/709217

**Published:** 2014-11-06

**Authors:** Pedro Boal Carvalho, Bruno Rosa, Maria João Moreira, José Cotter

**Affiliations:** Centro Hospitalar do Alto Ave, Rua dos Cutileiros, Creixomil, 4831-044 Guimarães, Portugal

## Abstract

*Objectives.* Small bowel capsule endoscopy (SBCE) plays a decisive role in the obscure gastrointestinal bleeding (OGIB) diagnosis. Antithrombotics may increase bleeding risk in patients with preexistent lesions or through direct mucosal aggression. We aimed to correlate antithrombotics usage with lesions with bleeding potential found in SBCE. *Methods.* Retrospective single-center study including 274 consecutive SBCE performed over 7 years for OGIB. The lesions were classified as P0 (no bleeding potential), P1 (uncertain bleeding potential: erosions), and P2 (high bleeding potential: angioectasias, ulcers, and tumors). We assessed antiplatelet and anticoagulant drug use during the 60 days preceding SBCE. *Results.* One-third of the patients were under antithrombotic therapy. The diagnostic yield of SBCE for P2 lesions was 30.0%. Angioectasias (20.4%) were the most frequently observed lesions. There was a significant correlation between anticoagulant drug use and a higher incidence of P2 lesions in the small bowel (43.2% versus 26.5%; OR = 2.11, *P* = 0.026). We found no significant correlation between antiplatelets and lesions with bleeding potential in SBCE. *Conclusions.* Small bowel lesions with high bleeding potential were more frequently detected when the patient was on anticoagulant drugs, resulting in a twofold risk. Antiplatelet drugs were not associated with small bowel lesions.

## 1. Introduction

Obscure gastrointestinal bleeding (OGIB), defined as bleeding of unknown origin that persists or recurs after negative esophagogastroduodenoscopy (EGD) and colonoscopy [1], is responsible for 5% of all gastrointestinal hemorrhages [2]. The small bowel is the leading source of OGIB, representing up to 75% of the cases [1]. The diagnosis and treatment of OGIB remained, therefore, a challenge to clinicians, but recent techniques, such as small bowel capsule endoscopy (SBCE), have unveiled the small bowel and contributed to improvement of its clinical management. In patients presenting with OGIB, SBCE diagnostic yield ranges from 38 to 93% [3–7], superior to other diagnostic modalities—push enteroscopy, computed tomography, and angiography [3, 8]. Thus, SBCE is currently considered the first-line examination for patients presenting with OGIB, both visible (hematochezia or melena) and occult (iron deficient anemia or positive fecal occult blood test (FOBT)) [9].

Nonsteroidal anti-inflammatory drugs (NSAIDs) and antiplatelet drugs are some of the most prescribed drugs worldwide. There were over 43 million regular aspirin users in 2010 in the USA alone, one-fifth of the adult population, showing an upward trend [10].

NSAID injury to the small bowel, resulting in mucosal erosions, ulcers, and ultimately scarring, is more common than NSAID-associated gastropathy [11] but was only fully appreciated on the advent of SBCE [12]. NSAID enteropathy is now a popular topic, as it represents one of the most common causes of OGIB [13, 14]. The evidence regarding the effects of antiplatelet drugs on the small bowel mucosa is, on the other hand, still scarce. Both Smecuol et al. and Endo et al. found an increase in mucosal damage after two weeks of aspirin therapy on small groups of healthy volunteers [15, 16], and Shiotani et al., studying patients referred for OGIB, reported a significantly higher incidence of erosions and ulcers among patients treated with a combination of aspirin and thienopyridine [17]. Other authors, however, have found aspirin to be less aggressive to the small bowel mucosa than other NSAIDs [18].

Anticoagulants such as coumarins were previously associated with a sevenfold relative risk for gastrointestinal bleeding [19] and connected with up to 25% of such bleeding episodes [20]. Besides reports of small bowel hematomas [21–23], there was no published work reporting evidence of anticoagulant-associated lesions in the small bowel [17, 24]. Nevertheless, some authors have described an increased relative risk for recurrent OGIB in patients under anticoagulant therapy [25–27].

The aim of this study was to investigate whether SBCE findings could be associated with antithrombotic (both antiplatelet and anticoagulant) drug usage in patients with OGIB.

## 2. Methods

We performed a retrospective single-center study including all patients presenting with OGIB who underwent SBCE in our department during a 7-year period (between January 2007 and December 2013). All patients had written informed consent for SBCE examination. In every patient, an EGD and colonoscopy were performed, prior to the SBCE (interval <6 months), which were nondiagnostic.

OGIB was classified as visible when the patient presented with either melena or hematochezia and occult if there was iron deficient anemia (hemoglobin <13 g/dL for men, <12 g/dL for women) or a positive FOBT.

According to department protocol, patient drug use and relevant medical history are registered on a checklist prior to the SBCE procedure. We analyzed antithrombotic drug use, both antiplatelet drugs (low-dose aspirin and thienopyridines) and anticoagulant drugs (warfarin and low molecular weight heparin (LMWH)) as well as NSAID use and past medical history: diabetes mellitus, arterial hypertension, chronic kidney disease (defined as a creatinine clearance rate <60 mL/min/m^2^), and ischaemic heart disease.

NSAID intake within 2 months of SBCE was defined as exclusion criteria. The PillCam SB1 capsule (Given Imaging Ltd., Israel) was used from January to December 2007 and the PillCam SB2 capsule (Given Imaging Ltd., Israel) from January 2008 until December 2013. Patients were instructed to ingest only clear liquids on the day prior to the exam as well as adhere to a 12-hour fast; no additional bowel preparation was employed. Domperidone was used (10 mg) if the SBCE remained in the stomach for over 1 h (assessed through real-time viewing, Given Imaging) [28].

All SBCE were independently reviewed by 2 experienced gastroenterologists and discussed until a consensus was reached when discrepancies in the interpretation occurred.

Small bowel lesions were described using the commonly employed classification of Saurin et al. [29], as P0 (no bleeding potential, such as nodules and lymphangiectasias), P1 (uncertain bleeding potential, such as red spots or small erosions), and P2 (high bleeding potential, such as angioectasias, ulcers, tumors, or varices).

Statistical analysis was performed using SPSS 21.1 (WinWrap Basic). Univariate analyses were performed, using independent samples *t*-test for continuous variables and the *χ* ^2^ or Fisher's exact tests for categorical variables. Statistical significance was defined for *P* value < 0.05.

## 3. Results

A total of 302 patients underwent SBCE in our department for OGIB from January 2007 to December 2013. Of those, 28 were excluded for reporting NSAID consumption up to 2 months before the examination.

The clinical baseline characteristics of the 274 included patients are summarized in Table 1, as well as the reported antithrombotic drug use. Only 23 patients had normal hemoglobin levels (22 of them referred for positive FOBT and 1 of them for visible OGIB). More than half of the patients undergoing SBCE (62.4%; *n* = 164) were diagnosed with at least one comorbidity. We observed no significant association between OGIB presentation, gender, age, or comorbidity and the lesions found during SBCE.

More than one-third (38.0%, *N* = 104) of the patients were being treated with an antithrombotic drug, the majority of which with low-dose aspirin; only four patients were under dual antiplatelet treatment (low-dose aspirin plus clopidogrel). Of the 25 patients treated with anticoagulants alone, 15 were on LMWH and 16 were on warfarin. Thirteen patients were treated with both an antiplatelet agent (in all cases low-dose aspirin) and an anticoagulant (LMWH in 4 patients, warfarin in 9).

The use of antiplatelet drugs was significantly more frequent among older patients and patients with severe anemia, suffering from diabetes mellitus, arterial hypertension, and ischaemic heart disease. Anticoagulant use was significantly associated with older age, lower hemoglobin values, arterial hypertension, and ischaemic heart disease. Patients' characteristics regarding antithrombotic use are summarized in Table 2.

OGIB had a visible presentation in 49 (17.9%) patients. There was no significant association between the use of either antiplatelet drugs (17.8% versus 17.9%, *P* = 0.984) or anticoagulants (22.7% versus 17.0%, *P* = 0.360) and a visible OGIB presentation.

Overall, the diagnostic yield of SBCE for P2 lesions was 30.0% (*n* = 82). Among the patients with lesions with bleeding potential (P2 lesions) in the small bowel, the most frequent findings were angioectasias (20.4%; *n* = 56); less common findings included ulcers (5.8%; *n* = 16), tumors (3.3%; *n* = 9), and phlebectasias (0.4%; *n* = 1). Erosions were found in 19.3% (*n* = 53), while no lesions were found in 50.7% of the patients (*n* = 139).

**Figure 1:**
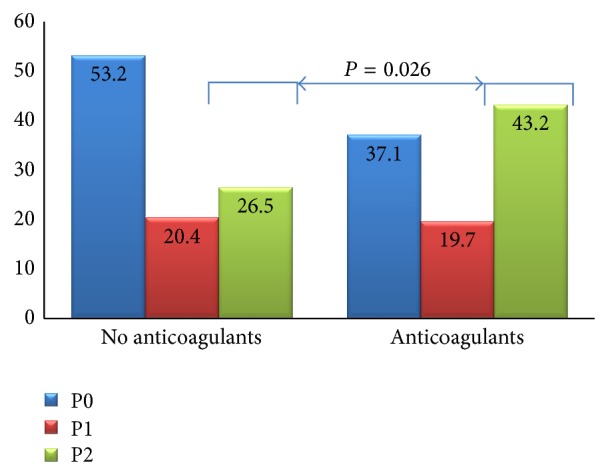
Prevalence (%) of SBCE bleeding lesions with and without anticoagulant drugs.

**Figure 2:**
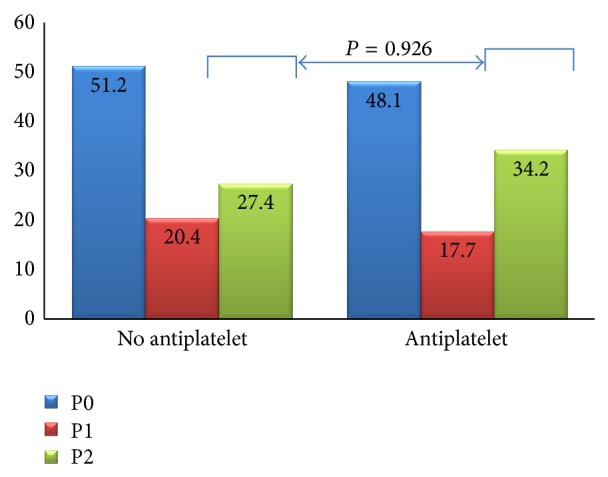
Prevalence (%) of SBCE bleeding lesions with and without antiplatelet drugs.

No differences were found regarding the detection of lesions with bleeding potential (P2) between patients taking antiplatelet drugs versus those with no blood thinning drugs (34.2 versus 27.4%, *P* = 0.268). However, anticoagulants were significantly associated with a higher prevalence of P2 lesions in the small bowel (43.2% versus 26.5%; OR = 2.11, *P* = 0.026). We performed a subanalysis on patients treated with both antiplatelet and anticoagulant drugs and found no significant association either with P2 lesions in general or with any individual finding. The incidence of SBCE bleeding lesions in patients medicated with antiplatelet and anticoagulant drugs is presented in Figures 1 and 2, respectively.

Small bowel tumors were significantly more prevalent among anticoagulant users (11.4% versus 1.7%, *P* = 0.002).  Table 3 summarizes the individual lesions found during SBCE under both antiplatelet and anticoagulant therapy.

## 4. Discussion

Despite being associated for decades with an increased risk of gastric ulcers [30, 31], aspirin's role in small bowel mucosal injury was only recently revealed. Smecuol et al. and Endo et al. both carried out prospective studies in small groups of volunteers under aspirin therapy [15, 16]. Endo et al. [16] found an increase in mucosal damage (30 versus 0%) in aspirin users, although the difference was not significant (*P* = 0.211). Smecuol et al. [15], employing small bowel permeability tests, also detected meaningful mucosal changes after just 14 days of low-dose aspirin therapy. Recently, Ehrhard et al. [32] studied 75 patients undergoing SBCE for OGIB and found a significant increase (*P* < 0.001) in mucosal breaks in patients receiving low-dose aspirin (71%) compared to those in the anticoagulant (20.0%) and control groups (12.5%).

Other antiplatelet drugs, on the other hand, were only associated with small bowel lesions in small studies and always in combined therapy with aspirin [17, 33]; Shiotani et al. reported a prevalence of 46% in small bowel lesions among patients treated with both low-dose aspirin and clopidogrel, significantly (*P* = 0.01) superior to either drug alone and to anticoagulant therapy [17].

In our series, antiplatelet drugs were not significantly associated with P2 lesions in the small bowel. These results are in contrast to the ones reported by the previously mentioned authors; however, van Weyenberg et al. [26] found no increase in small bowel erosions or ulcers in patients with continued antiplatelet drug therapy, suggesting that aggravating bleeding from preexisting mucosal lesions, and not direct mucosal injury, may be the crucial factor in clinically relevant OGIB.

Regarding the use of anticoagulants, some authors reported provocative gastrointestinal bleeding using low molecular weight heparin [24, 34]. Indirect evidence substantiating the importance for anticoagulants regarding small bowel bleeding can also be found in the work of both Koh et al. [4] and Kim et al. [27]: Koh et al. recently reported a significant risk increase of 5.02 (*P* = 0.007) for rebleeding in patients undergoing SBCE for OGIB under anticoagulant therapy; Kim et al., analyzing risk factors for rebleeding in negative SBCE, identified warfarin use as an independent risk factor (*P* = 0.001).

We report for the first time in the literature a significant association between anticoagulant use and an increased likelihood of finding a potentially bleeding lesion in the small bowel with capsule endoscopy (43.2% versus 26.5%), with a twofold risk (OR = 2.11, *P* = 0.026). Additionally, we found a significant increase in the yield of small bowel tumors in patients taking anticoagulant drugs; despite the possibility that anticoagulants may heighten these previously silent lesions, the small sample size allows for a type 1 error. Although this is a retrospective study performed in a single center, it has the merit of evaluating a large prospectively collected database, allowing for the analysis of the specific subset of patients with OGIB under anticoagulant therapy.

In conclusion, antiplatelet drugs, very prevalent in our series, did not influence OGIB presentation and were not significantly associated with lesions with high bleeding potential in the small bowel. In contrast, anticoagulants were significantly associated with a higher prevalence of P2 lesions in the small bowel; particularly, the diagnosis of small bowel tumors was significantly more frequent in patients under anticoagulant therapy.

These results highlight the importance of thoroughly investigating such patients when presenting with visible or occult gastrointestinal bleeding. Further prospective studies including patients with prescribed anticoagulants are warranted to confirm our findings.

## Figures and Tables

**Table 1:** Baseline patients' characteristics and antithrombotic use.

Characteristics, *n* (%)	
Age (y), median (range)	61.9 (19–91)
Female	170 (62.0)
Occult OGIB	225 (82.1)
Visible OGIB	49 (17.9)
Hemoglobin (g/dL), median (range)	9.3 (4.2–15.1)
Diabetes mellitus	79 (28.8)
Chronic kidney disease	29 (10.6)
Arterial hypertension	140 (51.1)
Ischaemic heart disease	54 (19.7)

Antithrombotic drug use, *n* (%)	

Antithrombotics	104 (38.0)
Antiplatelet alone	60 (21.9)
Aspirin	45 (16.4)
Thienopyridine	11 (4.0)
Both	4 (1.5)
Anticoagulant alone	31 (11.3)
Heparin/LMWH	15 (5.4)
Warfarin/acenocoumarol	16 (5.8)
Both	13 (4.7)

**Table 2:** Baseline characteristics of patients under antiplatelet and anticoagulant drugs.

Characteristics	No antithrombotic	Antiplatelet user^1^	Anticoagulant user^1^
	(*n* = 170)	(*n* = 73)	(*n* = 44)
Age, y, mean (SD)	56.1 (18.7)	72.40 (12.7)^2^	71.8 (13.4)^2^
Female sex, *n* (%)	109 (64.1)	44 (60.3)	22 (50.0)
Visible OGIB, *n* (%)	30 (17.6)	13 (17.8)	10 (22.7)
Hemoglobin, g/dL (SD)	9.7 (2.1)	8.7 (1.9)^2^	8.7 (1.8)^2^
Comorbidity			
Diabetes mellitus, *n* (%)	34 (20.0)	36 (49.3)^2^	17 (38.6)
Arterial hypertension, *n* (%)	69 (40.6)	53 (72.6)^2^	29 (65.9)^2^
Ischaemic heart disease, *n* (%)	22 (12.9)	22 (30.1)^2^	17 (38.6)^2^
Chronic kidney disease, *n* (%)	14 (8.2)	10 (13.7)	8 (18.2)

^1^Thirteen patients were treated with both antiplatelet and anticoagulant drugs.

^2^ *P* < 0.05.

**Table 3:** SBCE findings in patients under antiplatelet and anticoagulant drugs.

	Antiplatelet			Anticoagulant		
	No	Yes	*P*	No	Yes	*P*
	(*n* = 201)	(*n* = 73)		(*n* = 230)	(*n* = 44)	
Erosions, *n* (%)	40 (19.9)	14 (19.2)	n.s	46 (20.0)	8 (18.2)	n.s
Ulcers, *n* (%)	11 (5.5)	5 (6.8)	n.s	12 (5.2)	4 (9.1)	n.s
Angioectasias, *n* (%)	39 (19.4)	17 (23.3)	n.s	43 (18.7)	13 (29.5)	n.s
Tumors, *n* (%)	7 (3.5)	2 (2.7)	n.s	**4 (1.7)**	**5 (11.4)**	**0.002**
